# Sequence-specific Recruitment of Heterochromatin Protein 1 via Interaction with Krüppel-like Factor 11, a Human Transcription Factor Involved in Tumor Suppression and Metabolic Diseases*

**DOI:** 10.1074/jbc.M112.342634

**Published:** 2020-12-18

**Authors:** Gwen Lomberk, Angela J. Mathison, Adrienne Grzenda, Seungmae Seo, Cathrine J. DeMars, Sumera I. Ilyas, Juliana Bonilla-Velez, Ezequiel Calvo, Martin E. Fernandez-Zapico, Juan Iovanna, Navtej S. Buttar, Raul Urrutia

**Affiliations:** ‡ Department of Biochemistry and Molecular Biology, Laboratory of Epigenetics and Chromatin Dynamics, GIH Division; ¶ Schulze Center for Novel Therapeutics, Mayo Clinic, Rochester, Minnesota 55905; § Molecular Endocrinology and Oncology Research Center, CHUL Research Center, Quebec 61V 462, Canada; ‖ INSERM U.624, Parc Scientifique et Technologique de Luminy, Marseille F-13288, France

**Keywords:** Chromatin, Coregulator Transcription, Cxcr4, Krüppel-like Factor (KLF), Transcription Regulation, Heterochromatin Protein 1 (HP1)

## Abstract

Heterochromatin protein 1 (HP1) proteins are “gatekeepers” of epigenetic gene silencing that is mediated by lysine 9 of histone H3 methylation (H3K9me). Current knowledge supports a paradigm whereby HP1 proteins achieve repression by binding to H3K9me marks and interacting to H3K9 histone methyltransferases (HMTs), such as SUV39H1, which methylate this residue on adjacent nucleosomes thereby compacting chromatin and silencing gene expression. However, the mechanism underlying the recruitment of this epigenetic regulator to target gene promoters remains poorly characterized. In the current study, we reveal for the first time a mechanism whereby HP1 is recruited to promoters by a well characterized Krüppel-like transcription factor (KLF), in a sequence-specific manner, to mediate complex biological phenomena. A P*X*V*X*L HP1-interacting domain identified at position 487–491 of KLF11 mediates the binding of HP1α and KLF11 *in vitro* and in cultured cells. KLF11 also recruits HP1α and its histone methyltransferase, SUV39H1, to promoters to limit KLF11-mediated gene activation. Indeed, a KLF11ΔHP1 mutant derepresses KLF11-regulated cancer genes, by inhibiting HP1-SUV39H1 recruitment, decreasing H3K9me3, while increasing activation-associated marks. Biologically, impairment of KLF11-mediated HP1-HMT recruitment abolishes tumor suppression, providing direct evidence that HP1-HMTs act in a sequence-specific manner to achieve this function rather than its well characterized binding to methylated chromatin without intermediary. Collectively, these studies reveal a novel role for HP1 as a cofactor in tumor suppression, expand our mechanistic understanding of a KLF associated to human disease, and outline cellular and biochemical mechanisms underlying this phenomenon, increasing the specificity of targeting HP1-HMT complexes to gene promoters.

## Teaser

**Background:** Chromatin remodeling mechanisms utilized by Krüppel-like factor proteins remain poorly defined.

**Results:** Krüppel-like factor 11 directly recruits heterochromatin protein 1α to promoters in a sequence-specific manner.

**Conclusion:** Coupling to heterochromatin protein 1 α and its histone methyltransferase underlies Krüppel-like factor-mediated gene expression and tumor suppression.

**Significance:** This data advances our understanding of how chromatin-mediated mechanisms achieve these functions with increased specificity for target genes.

## Introduction

Heterochromatin protein 1 (HP1)^3^ was first discovered in *Drosophila* as a dominant suppressor of position effect variegation and major component of heterochromatin (1, 2). These non-histone chromatin proteins comprise an evolutionarily conserved family of epigenetic regulators involved in the establishment and maintenance of higher order chromatin structures. Belonging to the superfamily of chromodomain-containing proteins, the HP1 chromodomain binds to methylated lysine 9 of histone H3 (H3K9me) (3, 4). However, HP1 proteins define their own subfamily within this superfamily due to the presence of a second unique conserved domain in the carboxyl-half of the protein, the chromo shadow domain, which functions in homo/heterodimerization and interaction with other proteins (5). These two domains are separated by a less conserved linker region, the most variable amino acid sequence among HP1 proteins that is highly amenable to post-translational modifications, especially phosphorylation (6, 7, 8, 9). HP1 remodels chromatin through interactions with HP1-binding proteins containing a consensus sequence, P*X*V*X*L (10), or in a P*X*V*X*L-independent manner (8, 11, 12). Interestingly, these various interactions can occur in an HP1 isoform-specific manner or universally with all three isoforms, and may depend on particular post-translational modifications and involve proteins with a wide variety of cellular functions (13). Thus, HP1 is implicated in the regulation of an extensive variety of nuclear processes.

Current knowledge supports a paradigm whereby HP1 proteins repress gene expression by binding to H3K9me marks and interacting to H3K9 HMTs, such as G9a or SUV39H1, which methylate this residue on adjacent nucleosomes thereby compacting chromatin and silencing gene expression (14, 15). Thus far, this phenomenon has primarily been assumed to occur independently of sequence-specific DNA sites. In the current study, we examined whether sequence-specific recruitment of HP1 to promoters occurs to mediate complex biological phenomena. We strategically chose Krüppel-like factor 11 (KLF11) for our model, as this sequence-specific transcription factor and tumor suppressor protein contains a consensus P*X*V*X*L HP1-interacting domain within its C terminus, which we confirm is functional *in vitro* and *in vivo*. Recently, KLF11 has elicited significant attention due to its roles in tumorigenesis, cholesterol metabolism, erythropoiesis, and diabetes (16, 17, 18, 19, 20). KLF11 belongs to the KLF family of transcription factors, which are structurally defined by the presence of three highly homologous, C-terminal Cys_2_-His_2_ zinc finger DNA binding domains and a variant N-terminal domain that contain transcriptional regulatory motifs (21). KLF proteins bind to similar, yet distinct GC-rich target sequences, and some function as activators, others act as repressors, whereas yet another subset can function as either an activator or repressor depending on the promoter context. Interestingly, KLF sites have been previously shown to function as polycomb response elements (PREs) in *Drosophila* (22, 23), suggesting their potential to regulate the sequence-specific recruitment of HMT complexes. Interestingly, we find that this KLF transcription factor recruits the HP1-SUV39H1 HMT system to target promoters in a sequence-specific manner rather than through the canonical binding of HP1 to H3K9me3 without intermediaries. Moreover, our results demonstrate the importance of this novel mechanism in regulating both gene expression and several important cellular functions associated to tumor suppression. Thus, these studies expand our understanding on how HP1-HMT containing complexes are targeted to a specific *cis*-regulatory genomic site to regulate chromatin dynamics, gene expression, and tumor suppression.

## EXPERIMENTAL PROCEDURES

##### Tissue Culture and Reagents

Cell lines were obtained from the American Type Culture Collection (ATCC, Manassas, VA). Cells were cultured and transiently transfected as described previously (19, 24).

##### Plasmids, siRNA, and Recombinant Adenovirus

Standard molecular biology techniques were used to clone full-length KLF11, as well as specified KLF11 deletions into pcDNA3.1/His (Invitrogen), pCMV-Tag 2B (Stratagene, La Jolla, CA), and EYFP vectors; HP1α, HP1β, and HP1γ cDNAs were cloned into pGEX (GE Healthcare) and EYFP vectors, as well as the *CXCR4* promoter into pGL3 (Promega) as previously described (8, 25). QuikChange® Site-directed Mutagenesis was performed as suggested by the manufacturer (Agilent Technologies, Inc., Santa Clara, CA). For HP1α-specific shRNA, complementary oligonucleotides were synthesized for the target sequence, annealed, and ligated into the pCMS3 vector (kindly provided by Dr. Daniel Billadeau, Mayo Clinic, Rochester, MN). The hTERT promoter luciferase construct was kindly provided by Dr. Silvia Bacchetti (McMaster University). All constructs were verified by sequencing at the Mayo Clinic Molecular Biology Core Facility. HP1α (*CBX5*) ON-TARGETplus SMARTpool siRNA was purchased from Thermo Scientific-Dharmacon (Lafayette, CO). Epitope-tagged (His_6_-Xpress^TM^) KLF11 and KLF11ΔHP1, as well as empty vector (Ad5CMV), were generated as recombinant adenovirus in collaboration with the Gene Transfer Vector Core at the University of Iowa.

##### GST Pulldown Assays, Immunoprecipitation, and Western Blot

GST and GST fusion protein purification, *in vitro* translation, GST pulldown assays, immunoprecipitation, and Western blot were all done as previously described (8). Antibodies were used against the FLAG (Sigma) or His tags (OMNI D8; Santa Cruz Biotechnology, Santa Cruz, CA) to detect recombinantly expressed KLF11 or KLF11ΔHP1, HP1α (Millipore), Sin3a (Santa Cruz Biotechnology), or anti-p300 (Millipore). All histone mark and CXCR4 antibodies were obtained from Abcam (Cambridge, MA), anti-SUV39H1 was from Millipore, and caspase-3 antibodies were obtained from Cell Signaling Technology (Danvers, MA).

##### Bimolecular Fluorescence Complementation (BiFC)

Cells were co-transfected with the EYFP(1) and EYFP(2) expression vectors and after 48 h, examined by confocal microscopy, as described previously (8).

##### Electrophoretic Mobility Shift Assays (EMSA)

Gel shift assays were performed as described (26). Annealed, double-stranded 34-bp oligonucleotides encompassing potential KLF sites in the human *CXCR4* promoter and mutated site 3 were end labeled with [γ-^32^P]ATP using T4 polynucleotide kinase as indicated by the manufacturer (Promega). Samples were loaded immediately onto a 4% nondenaturing polyacrylamide gel, run for 2 h at 200 V at room temperature, vacuum-dried, and exposed to HyBlot CL^TM^ autoradiography film (Denville Scientific Inc., Metuchen, NJ).

##### Hoescht Staining

Using Hoechst 33342 stain, characteristic nuclear apoptotic changes such as nuclear fragmentation, chromatin condensation, and margination were determined as previously described (24, 27). At least 200 Panc1 cells in six different high-power fields were counted for each individual time point. Each experiment was performed in triplicate. Results were expressed as mean ± S.E., and statistical analyses were performed using a Student's *t* test.

##### β-Galactosidase Staining

Senescence-associated β-galactosidase activity was detected in primary fibroblast cells using the Senescence Cells Histochemical Staining Kit (Sigma), according to the manufacturer's instructions. Each experiment was performed in triplicate. Results were expressed as mean ± S.E., and statistical analyses were performed using a Student's *t* test.

##### Reporter Assays

hTERT promoter activity was monitored via reporter assay as previously described (28). CHO cells (3 × 10^5^) were transfected in 6-well plates by Lipofectamine^TM^ (Invitrogen) according to the manufacturer's recommendations. For *CXCR4* promoter activity, reporter assays were performed in Panc1 cells, as described (19). Proteins were isolated 48 h post-transfection and the relative luciferase expression was assayed using the Luciferase Assay System (Promega) and a Turner 20/20 luminometer. In all experiments, luciferase activity was controlled by total protein concentration. Each experiment was performed at least three different times in triplicate, expressed as mean ± S.E., and statistical analyses were performed using a Student's *t* test.

##### Foci Formation and Soft Agar Assays

Oncogenic KRAS foci formation assays in NIH/3T3 cells and soft agar assays in Panc1 cells were performed as previously described (19). Each experimental condition was performed three independent times at least in triplicate. Results were expressed as mean ± S.E., and statistical analyses were performed using a Student's *t* test.

##### Tumor Xenografts

4 × 10^6^ L3.6 and Panc1 cells were injected subcutaneously into the hind leg of female athymic nude mice, and tumor growth was measured weekly. We estimated tumor volume from tumor length (l) and width (w) using the formula: 4/3 × π × [(l + w)/4]^3^ (29). Animal experiments were approved by the Institutional Animal Care and Use Committee at Mayo Clinic College of Medicine. Results were expressed as mean ± S.D., and statistical analyses were performed using a Student's *t* test.

##### Chromatin Immunoprecipitation Assay (ChIP)

ChIP assay was performed as previously described (27). Sequential ChIP was performed with the Re-ChIP-IT® kit (Active Motif, Carlsbad, CA) according to the manufacturer's recommendations. Specific primers for 154- and 263-bp regions of the *CXCR4* promoter at the KLF11 site and directly 5′ to the site, respectively, were utilized for PCR amplification (at KLF site: 5′-CCTCCTTCCTCGCGTCTG-3′, 5′-CGTCACTTTGCTACCTGCTG-3′; 5′ to KLF site: 5′-TTTACCTCCTGAATGGGCTG-3′, 5′-TCCTAAGTTTGAGGGAAGCG-3′).

##### Microarray and RT-PCR Validation

Global Expression Profiling (Affymetrix Human Gene 1.0 ST arrays with 28,869 well annotated genes and 764,885 distinct probes) was carried out at the Microarrays Facility of the Research Center of Laval University CRCHUL. Intensity files were generated by Affymetrix GCS 3000 7G and the Gene-Chip Operating Software (Affymetrix, Santa Clara, CA). Data analysis, background subtraction, and intensity normalization were performed using Robust Multiarray Analysis (30). Differentially expressed genes and false discovery rate were estimated from *t* test (>0.005) and corrected using Bayes approach (31, 32). Data analysis, hierarchical clustering, and ontology was performed using the OneChanelGUI to extend affylmGUI graphical interface capabilities (33), and Partek Genomics Suite, version 6.5 (Partek Inc., St. Louis, MO) with analysis of variance analysis. RT-PCR was performed as previously described (34). Specific primer sequences are available upon request to authors.

## RESULTS

##### HP1 Interacts with the Sequence-specific Tumor Suppressor, KLF11, via a PXVXL Motif

**FIGURE 1:**
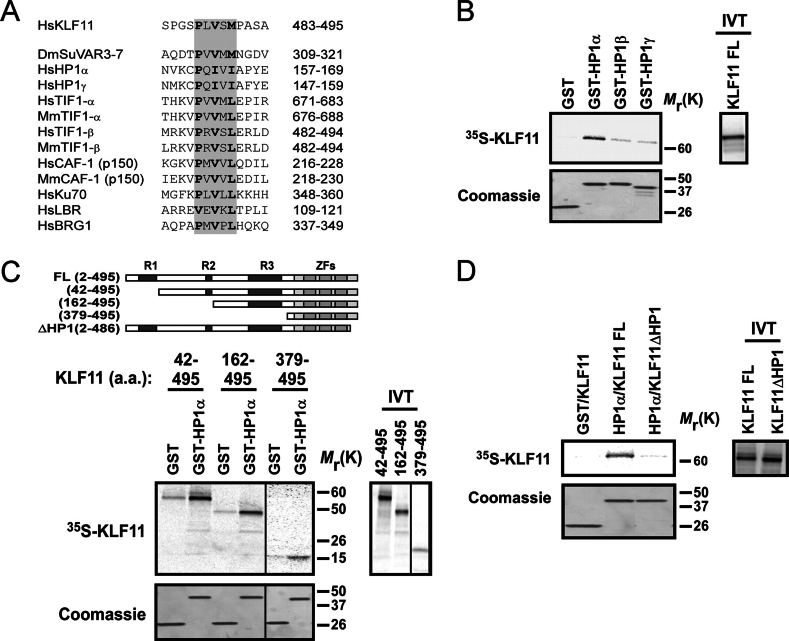
**HP1 interacts with the sequence-specific tumor suppressor, KLF11 *in vitro*.***A*, KLF11 contains a P*X*V*X*(L/M) domain. HP1-interacting protein sequences containing the pentameric consensus (*top*, *gray-shaded regions*) align with the C terminus of KLF11. Amino acid positions are denoted to the *right. Dm*, *D. melanogaster*; *Mm*, *Mus muculus*; *Hs*, *Homo sapiens. B,* HP1 interacts with KLF11 *in vitro*. KLF11 interacts with all 3 HP1 isoforms with HP1α displaying the strongest binding. Phosphorimage results of ^35^S-KLF11 binding (*upper*) and Coomassie staining of GST-HP1 proteins as a loading control (*lower*) are shown. GST alone was included as a negative control. Input (10%) of the *in vitro* translated (*IVT*) protein is shown to the *right. C,* HP1 does not bind within the N-terminal domains of KLF11. GST-HP1α still interacted with KLF11 upon deletion of the R1 (42–495) or both, the R1 and R2 together along with the intervening sequence (162–495). GST-HP1α interacted with KLF11 (379–495), indicating that HP1 interacts with the C terminus of KLF11. Phosphorimage results of binding (*upper*) and loading control Coomassie staining of GST proteins (*lower*) are shown. GST alone was included as a negative control for each. Inputs (10%) of *in vitro* translated proteins are shown to the *right*. The schematic (*top*) depicts domains of KLF11, including the R1, R2, R3, and zinc finger (ZFs) domains. Various deletion mutants of KLF11, utilized for mapping, are shown. *D,* HP1 interacts with residues 487–495 of KLF11. Deletion KLF11 (486), or KLF11ΔHP1, abolished binding of HP1α, indicating that this P*X*V*X*(L/M) domain is the site of HP1 interaction. Phosphorimage results of binding (*upper*) and Coomassie staining of GST proteins for a loading control (*lower*) are shown. Inputs of *in vitro* translated proteins are shown to the *right*.

Bioinformatics-based analysis, using the Eukaryotic Linear Motif data base, demonstrated that KLF11, a well characterized sequence-specific transcription factor and tumor suppressor protein, possesses a potential binding site for HP1 (P*X*VX(L/M) domain) (Fig. 1*A*). If functional, this domain should recruit HP1-HMT complexes to *cis*-KLF promoter sequences. To test this hypothesis, we initially performed *in vitro* binding assays using ^35^S-KLF11 and the three human GST-HP1 proteins, HP1α, HP1β, and HP1γ. We demonstrate that KLF11 binds all three isoforms, with HP1α showing the strongest comparative binding (Fig. 1*B*). The location of this HP1 binding site was determined using several KLF11 mutants (Fig. 1*C*) along with HP1α, which had the most robust *in vitro* binding to KLF11. Deletion of the R1 and R2 domains of KLF11 (35), along with their linking sequences, still maintained binding to HP1 (amino acids 42–495 and 162–495, respectively). Furthermore, the C-terminal portion (amino acids 379–495) preserved HP1 binding (Fig. 1*C*), confirming that the HP1-binding site lies within this region. Deletion of the 9 most C-terminal amino acids, as shown with the KLF11 (amino acids 2–486) mutant, revealed that, indeed, the consensus P*X*V*X*(L/M) (amino acids 487–491) of KLF11 functions as a *bona fide* HP1 binding site (Fig. 1*D*), creating the KLF11ΔHP1 mutant. Together, our biochemical experiments demonstrate that KLF11 contains an HP1-binding domain and map this site at the precise amino acid level, suggesting that this transcription factor may anchor HP1 to promoters in a sequence-specific fashion.

**FIGURE 2:**
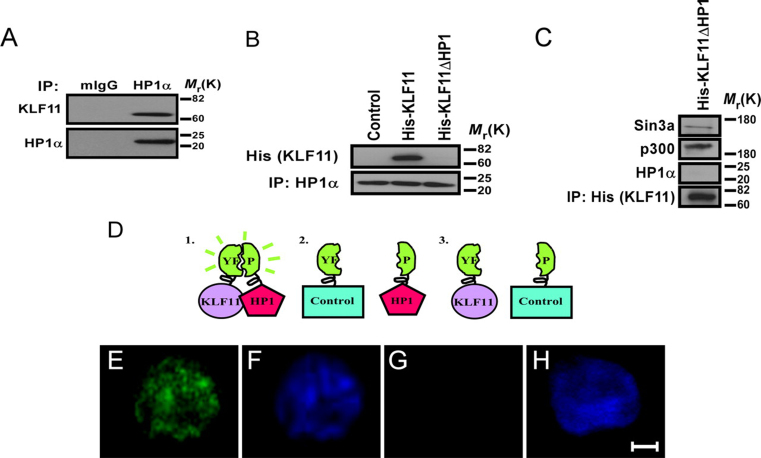
**HP1 binds to KLF11 *in vivo*.***A,* endogenous HP1α and KLF11 proteins interact in cells. Endogenous HP1α immunoprecipitates endogenous KLF11 from BxPC3 nuclear extracts. Upon immunoprecipitation with an HP1α-specific antibody, a Western blot was performed using a KLF11-specific antibody. Mouse IgG was used as a negative control. *B,* HP1α-KLF11 interaction in cells requires its C-terminal P*X*V*X*(L/M) motif. Immunoprecipitation (*IP*) with an HP1α-specific antibody (*lower*) demonstrates binding of His-tagged KLF11 WT (*upper*), but is disrupted with His-tagged KLF11ΔHP1. EV was used as a negative control. *C*, KLF11ΔHP1 does not impair binding to other KLF11 co-factors. Although KLF11ΔHP1 does not interact with HP1α, upon immunoprecipitation of the His-tagged deletion mutant, interactions with other KLF11 corepressors/coactivators, namely Sin3a and p300, are not disrupted, supporting mutant specificity. *D–H*, BiFC of HP1 and KLF11 demonstrates nuclear interaction. Models of predicted results are shown (*D*). Cotransfection of N-terminal EYFP protein (EYFP(1)) fused to KLF11 with the C-terminal EYFP (EYFP(2)) fused to HP1α demonstrates interaction in the nucleus through fluorescence reconstitution (*E*) and nuclear DAPI co-stain (*F*). A negative control leucine zipper protein fused to EYFP with either KLF11 (*G*) or HP1 (not shown) fused to their respective EYFP halves did not reconstitute fluorescence. DAPI staining is shown to visualize the nucleus (*H*). *Scale bar* represents 5 μm.

**FIGURE 4:**
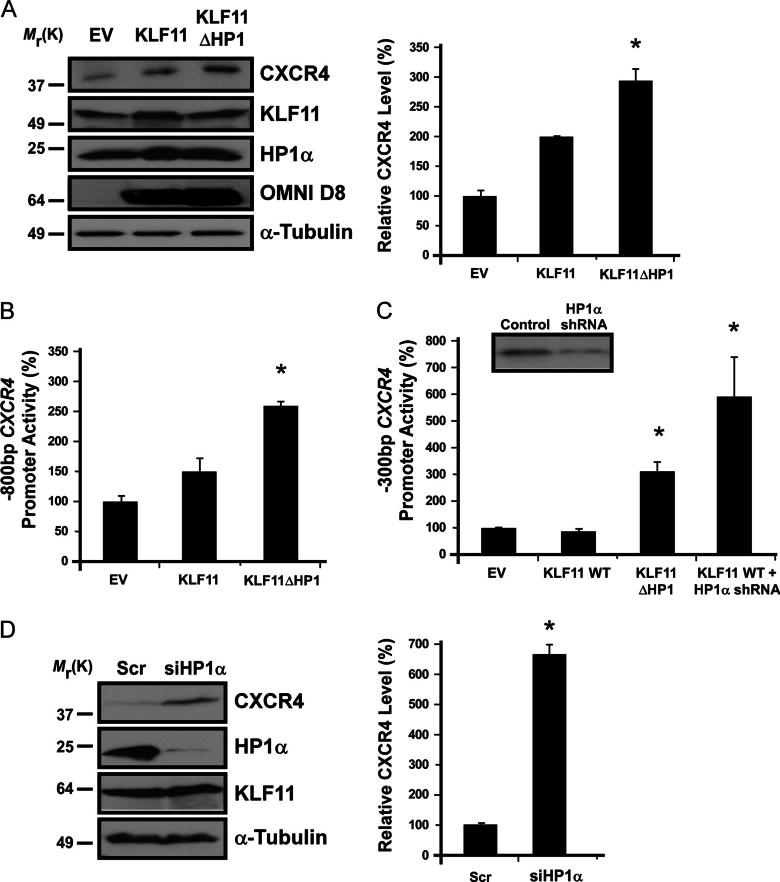
**Functional site in the *CXCR4* promoter is defined by HP1 recruitment in a sequence-specific manner by KLF11.***A,* KLF11ΔHP1 increases CXCR4 protein levels. Protein levels of CXCR4 corroborated the expression pattern observed by array and RT-PCR, consistent with derepression by KLF11ΔHP1. Endogenous levels of KLF11 and HP1α protein remained unchanged. α-Tubulin was used as a loading control. OMNI D8 antibody was used to detect ectopic His-tagged KLF11 WT and KLF11ΔHP1 as an expression control. Densitometric analysis expressed as CXCR4/α-tubulin is shown on the *right. Error bars* represent S.D. * denotes *p* < 0.05 for EV *versus* KLF11ΔHP1, as well as KLF11WT *versus* KLF11ΔHP1. *B,* KLFΔHP1 derepresses the −800-bp *CXCR4* promoter. A −800-bp fragment of the *CXCR4* promoter was utilized for luciferase-based reporter assays, validating the expression pattern observed by microarray, RT-PCR, and Western blot. KLF11ΔHP1 significantly derepressed *CXCR4* promoter activity, whereas KLF11 WT showed negligible changes. * denotes *p* < 0.05 for EV *versus* KLF11ΔHP1, as well as KLF11WT *versus* KLF11ΔHP1. *C*, the KLF-HP1 site is located within the −300-bp *CXCR4* promoter. Similar results to the −800-bp promoter were observed with a −300-bp promoter, indicating that the KLF-HP1 site is located within this smaller fragment of the promoter. KLF11 WT with knockdown of HP1α by shRNA effectively reproduced the derepression observed with KLF11ΔHP1. *Inset* shows a control Western blot of HP1α knockdown. The mean ± S.E. from at least three independent experiments performed in triplicate are shown for both reporters. * denotes *p* < 0.05. *D,* knockdown of HP1α results in increased CXCR4 protein. Similar to KLF11ΔHP1, siRNA-mediated knockdown of HP1α (siHP1α) increases CXCR4 at the protein level compared with scrambled siRNA control (*Scr*). HP1α levels are shown as control of efficient knockdown. Endogenous KLF11 protein levels remain unchanged, and α-tubulin was used as a loading control. Densitometric analysis expressed as CXCR4/α-tubulin is shown on the *right. Error bars* represent S.D. * denotes *p* < 0.05.

Subsequently, we investigated the formation of the KLF11-HP1 complex in living cells using two complementary techniques, co-immunoprecipitation and BiFC (8). Endogenous HP1α immunoprecipitated endogenous KLF11 protein from nuclear lysates of various epithelial cell lines, including BxPC3 (Fig. 2*A*), Panc1, and PL45 (data not shown). In addition, transfection of His-tagged KLF11 wild type (WT) or KLF11ΔHP1 confirmed that HP1α is able to immunoprecipitate KLF11 WT, but not KLF11ΔHP1 (Fig. 2*B*). KLF11ΔHP1 still bound to DNA (as shown below in Fig. 4*D*) and maintained interactions with other known KLF11 co-factors (Fig. 2*C*), including Sin3a (25) and p300 (36). Thus, KLF11ΔHP1 became a valuable tool for identifying processes that depend upon the sequence-specific recruitment of HP1 by KLF11. For visualizing subcellular localization of this interaction in living cells, we utilized BiFC by fusing the EYFP N terminus (EYFP(1)) to KLF11 and the EYFP C-terminal portion (EYFP(2)) to HP1α (Fig. 2*D*). Interaction was detected by yellow fluorescence reconstitution (Fig. 2*E*) and localized, as expected, in the nucleus (Fig. 2*F*). Negative controls co-expressing a leucine zipper protein with either KLF11 (Fig. 2, *G* and *H*) or HP1α (data not shown) fused to their respective EYFP halves did not reconstitute fluorescence, demonstrating the fluorescence obtained is specific and devoid of background. Combined, these experiments concordantly reveal the formation of a KLF11-HP1α complex in cells, confirming the binding of HP1 to KLF11 and providing helpful molecular information to understand the formation of this complex.

##### Sequence-specific Recruitment of HP1 by KLF11 Regulates the Expression of Cancer-associated Genes

**FIGURE 3:**
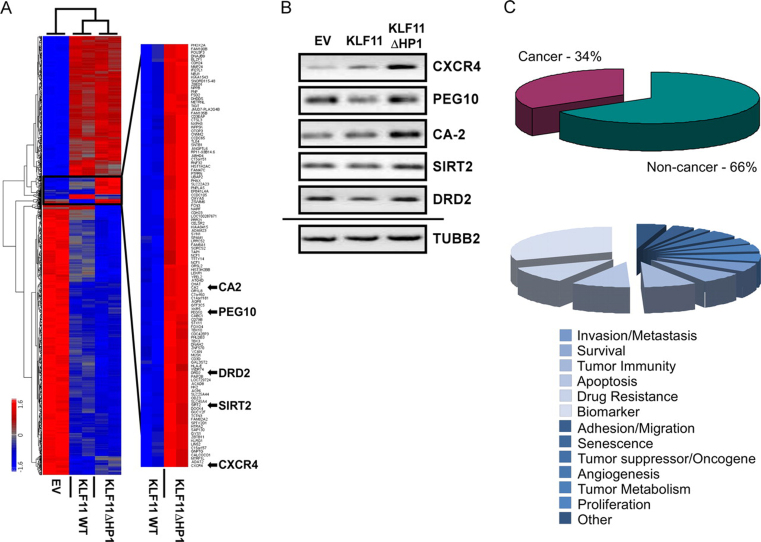
**Sequence-specific recruitment of HP1 by KLF11 regulates expression of cancer-associated genes.***A,* KLF11ΔHP1 displays a distinct pattern of gene expression. Heat map of microarray data is shown comparing the expression patterns from Panc1 cells infected with EV, KLF11 WT, or KLF11ΔHP1. *Inset* displays genes derepressed by KLF11ΔHP1. *B,* RT-PCR validates the expression pattern of select genes derepressed by KLF11ΔHP1. β2-Tubulin (*TUBB2*) was used as an internal control. *C,* KLF11ΔHP1 regulates a large number of cancer-associated genes. *Upper chart* depicts that 34% of genes derepressed by KLF11ΔHP1 are related to cancer-associated processes. A break-down of these cancer-associated processes is also shown (*lower chart*).

KLF11 is a tumor suppressor protein for several tumors (19, 27, 37, 38, 39, 40, 41, 42). Due to the established role of both KLF11 and HP1 in gene regulation, we reasoned that these proteins may exert a tumor suppressor function, at least in part, by modulating gene expression. We generated global expression profiles using the Affymetrix Human Gene 1.0 ST array from cells transduced with empty vector (EV) control, KLF11 WT, or KLF11ΔHP1 (Fig. 3*A*). Expression of epitope-tagged KLF11 WT and KLF11ΔHP1 was controlled by Western blot (suppemental Fig. S1). Normally, KLF11 results from the integration of the function of its corepressor and coactivator proteins that work together to maintain a steady-state level of expression for a particular promoter. Genes that require KLF11 interaction with HP1 for their regulation were readily derepressed by KLF11ΔHP1. This result demonstrates that HP1 functions to limit KLF11-mediated gene activation. Detailed statistical and bioinformatics analysis identified genes that are significantly associated to KLF11 expression (*p* < 0.002). As expected from its tumor suppressor role, KLF11 regulates a series of cancer-associated genes, including those for apoptosis (*AKT1*, *TP53*, *CASP9*), growth and migration (*CXCR4*, *SIRT2*), and epigenetic regulation (*HDAC1*, *HDAC3*, *HDAC6*), whereas other genes are less studied in this context. Clustering algorithms identified a subgroup of genes regulated by KLF11 in an HP1-dependent manner (Fig. 3*A*, inset, and supplemental TableS1). Array validation by RT-PCR of select genes is shown (Fig. 3*B*). Most likely, any overall effect of the KLF11-HP1 interaction (*e.g.* tumor suppression) results from the concerted function of many target genes identified here. Nevertheless, with the well established role of many of these co-regulated targets in cancer (34%; Fig. 3*C*), their silencing by HP1 via interaction with KLF11 is congruent with the role of this protein in cancer-related processes. Internal validation of these experiments was revealed by the fact that a discrete subset of KLF11-regulated genes (1022 in total) was altered with the KLF11ΔHP1 mutant (118; 11.5%). This specific KLF11ΔHP1 mutant should not affect the entire set of KLF11-regulated genes as KLF11 interacts with several other co-regulators, such as Sin3a and p300 (25, 36). Mechanistically, this data demonstrates that HP1 binding to KLF11 triggers a distinct transcriptional response, which includes many genes involved in cancer-associated events.

##### KLF11 Recruits HP1 to the CXCR4 Promoter in a Sequence-specific Manner

**FIGURE 5:**
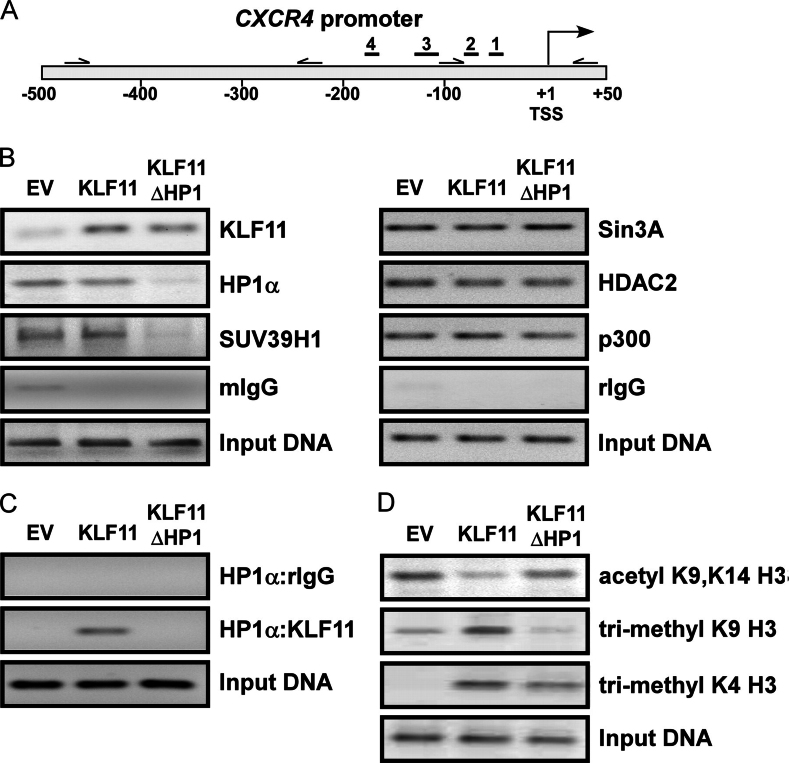
**KLF11 and HP1α occupy the *CXCR4* promoter *in vivo*.***A,* the *CXCR4* proximal promoter contains KLF sites. Map of the −500-bp proximal promoter of CXCR4 shows the relative location of 4 putative KLF sites (*solid lines labeled 1–4*). Numbering of the promoter is relative to the transcription start site (+1, *TSS*). *Half-arrows* indicate positions of the primer pairs used in ChIP experiments. *B,* HP1α and its HMT, SUV39H1, are recruited to the *CXCR4* promoter by KLF11. ChIP results demonstrate that in the presence of KLF11 WT, HP1α and SUV39H1 occupy the *CXCR4* promoter (*left*). However, replacement for KLF11ΔHP1 abolishes their recruitment. Recruitment of other KLF11 co-factors, Sin3A, HDAC2, and p300 remains unchanged (*right*). Mouse IgG (*mIgG*) and rabbit IgG (rIgG) serve as negative controls. Both KLF11 WT and ΔHP1 proteins bind to this promoter, confirming that KLF11ΔHP1 does not disrupt KLF11 promoter recognition and DNA binding. *C,* KLF11 and HP1α co-occupy the *CXCR4* promoter by sequential ChIP. Sequential ChIP experiments were performed in which chromatin-DNA complexes containing HP1α were immunoprecipitated in the first round and rIgG (HP1α:rIgG) or KLF11 (HP1α:KLF11) in the second round of immunoprecipitation. *CXCR4* was detected after sequential HP1α:KLF11 ChIP from KLF11 WT, but not KLF11ΔHP1-transfected cells, confirming that these two proteins co-occupy this region of the promoter. *D,* histone marks at the KLF-HP1 site change in a manner congruent with different promoter states. ChIP experiments were performed on the *CXCR4* promoter for acetyl-H3 (K9, K14), H3K4me3, and H3K9me3. KLF11 WT enriched the region with H3K9me3, but KLF11ΔHP1 lost its ability to support the writing of the H3K9me3 mark. The pattern of histone marks present at the *CXCR4* promoter predicts that KLF11ΔHP1 triggers a derepression event due to high levels of acetyl-H3 (K9 and K14) and H3K4me3 with concomitant loss of the H3K9me3 mark. Positive amplification of PCR products is shown in the *input DNA* lanes demonstrating that the region of the *CXCR4* promoter is present in all samples before immunoprecipitation.

The current dominant paradigm predicts that HP1 primarily regulates gene expression by globally binding to H3K9me3 with subsequent formation and spreading of heterochromatin (13). However, its binding to KLF11 led us to test an alternative mechanism, whereby KLF11 recruits HP1 directly to promoters in a sequence-specific manner. To test this hypothesis, we chose the *CXCR4* promoter as a model, which is derepressed by KLF11ΔHP1 (Fig. 3*A*), suggesting that HP1 recruitment to the promoter by KLF11 is necessary to limit gene expression. This is important as overexpression of *CXCR4* is associated with cancer development and metastasis (43). RT-PCR validated the *CXCR4* expression pattern observed by microarray (Fig. 3*B*). Furthermore, this result was confirmed at the protein level by Western blot (Fig. 4*A*; KLF11ΔHP1 2.94 ± 0.2-fold above EV, *p* < 0.05). Of note, endogenous levels of KLF11 and HP1α remained unchanged by transfection of the His-tagged KLF11 WT and KLF11ΔHP1 (Fig. 4*A*). To investigate promoter function, we first utilized a −800-bp fragment of the *CXCR4* promoter cloned upstream of luciferase cDNA for reporter assays. This −800-bp promoter validated the expression pattern observed by microarray, RT-PCR, and Western blot. Upon co-transfection of KLF11ΔHP1 with the *CXCR4* promoter reporter, luciferase activity was derepressed significantly (258 ± 9.4% normalized to EV, *p* < 0.05; Fig. 4*B*), whereas KLF11 WT showed negligible changes compared with EV (150 ± 24%, *p* > 0.05). Similar results were observed with a −300-bp promoter for KLF11ΔHP1 (306 ± 42.8% normalized to EV, *p* < 0.05; Fig. 4*C*) with no significant change for KLF11 WT (87.7 ± 10.5%, *p* > 0.05), indicating that the KLF11 site responsible for HP1 recruitment was indeed located within this smaller fragment of the promoter. Furthermore, shRNA-mediated knockdown of HP1α in the presence of KLF11 WT similarly derepressed promoter activity as KLF11ΔHP1 (587 ± 142.5% compared with EV, *p* < 0.05; Fig. 4*C*), further supporting the role of HP1 recruitment in the regulation of the *CXCR4* promoter. We further analyzed the effect of HP1α knockdown on CXCR4 at the protein level by Western blot. Similar to KLF11ΔHP1, knockdown of HP1α resulted in a significant increase in CXCR4 protein (668 ± 23.0% compared with control, *p* < 0.05; Fig. 4*D*). Knockdown of HP1α did not affect the level of endogenous KLF11 protein. To determine whether this effect reflected binding of both KLF11 and HP1 to the promoter, we designed primers within the −500-bp proximal *CXCR4* promoter (Fig. 5*A*) and performed ChIP assays. Indeed, HP1α was recruited to the *CXCR4* promoter by KLF11 WT but not KLF11ΔHP1 (Fig. 5*B*, *left*). Both KLF11 WT and ΔHP1 proteins bind to this promoter, confirming that deletion of the C-terminal HP1-binding site does not disrupt KLF11 promoter recognition and DNA binding (Fig. 5*B*, *left*). Notably, the recruitment of SUV39H1 was contingent on the integrity of the KLF11-HP1 complex because this HMT was absent with KLF11ΔHP1 (Fig. 5*B*, *left*). We found that recruitment of other KLF11 co-factors, including Sin3A, its catalytic partner HDAC2, as well as p300, remained unchanged (Fig. 5*B*, *right*). Furthermore, to substantiate that both KLF11 and HP1α definitively co-occupy the *CXCR4* promoter *in vivo* and that this co-occupation relies on an intact KLF11, we performed sequential ChIP analysis. For this purpose, we immunoprecipitated chromatin-DNA complexes containing HP1α in the first round and subsequently, KLF11 in the second round of immunoprecipitation. The *CXCR4* promoter was detected in the ChIP-enriched fragments from KLF11 WT, but not KLF11ΔHP1-transfected cells (Fig. 5*C*), confirming that these two proteins are indeed localized together on this promoter. Thus, combined, these results demonstrate that HP1α-SUV39H1 recruitment is dependent on KLF11 sequence-specific promoter binding and underscores the functionality of this recruitment in gene expression.

To further understand the functionality of KLF11-mediated HP1 recruitment, we investigated the promoter for histone marks to observe the associated state of chromatin remodeling events. The *CXCR4* promoter was occupied by acetylated H3 (K9 and K14) with both EV and KLF11ΔHP1, but levels were diminished with KLF11 WT (Fig. 5*D*). These marks are characteristically deposited by the histone acetyltransferases, p300, CBP, and PCAF, during gene activation (44). Interestingly, KLF11 enriched the promoter with H3K9me3, the mark recognized by HP1 and also deposited by its associated HMT, SUV39H1 (45), but KLF11ΔHP1 lost its ability to support the writing of the H3K9me3 mark on the promoter (Fig. 5*D*). Likely KLF11 recruits the HP1-SUV39H1 complex to maintain appropriate levels of expression while avoiding promoter dysregulation. This also provides evidence that the deposition of the H3K9me3 mark does not precede HP1 recruitment in this context, as typically believed, because in the absence of HP1 recruitment (KLF11ΔHP1) there is no H3K9me3 on the promoter. Finally, H3K4me3 was present in both KLF11 WT and ΔHP1, not discriminatory but serving as a marker poised for activation (Fig. 5*D*) (44). In summary, sequence-specific recruitment of HP1α via KLF11 is operational to mediate gene expression. Sequential ChIP experiments corroborate that these two proteins co-localize on the *CXCR4* promoter. Additionally, our ChIP experiments predict that KLF11ΔHP1 triggers a derepression event due to high levels of acetyl-H3 (K9 and K14) and H3K4me3 with concomitant loss of the H3K9me3 mark. This chromatin state on the *CXCR4* promoter coincides with the observed derepression of this gene. Thus, integration of these biochemical characteristics, namely expression levels, promoter assays, and chromatin marks, reveals a functional KLF11 site, which directs HP1α recruitment, present within the *CXCR4* promoter.

**FIGURE 6:**
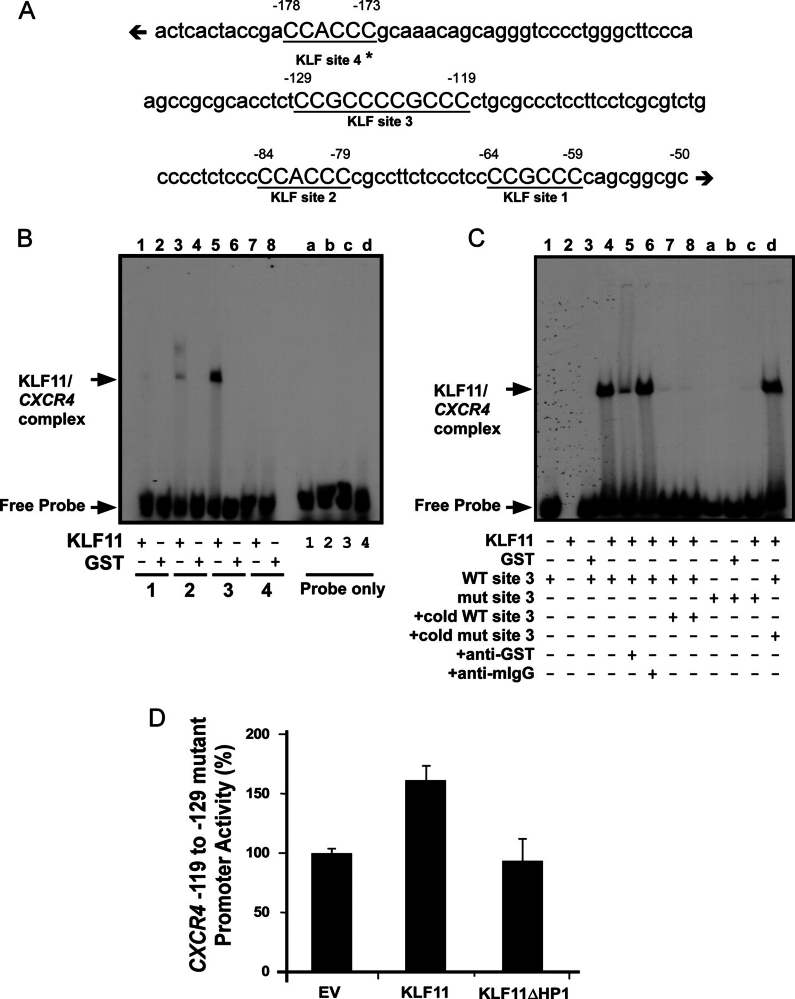
**The KLF site at −119 to −129 mediates HP1 recruitment to the *CXCR4* promoter.***A*, the *CXCR4* promoter has four putative KLF binding sites. The diagram represents a schematic of the human *CXCR4* proximal promoter (−50 to −190 relative to TSS) containing four KLF elements (*underlined*, 1–4 relative to TSS). Site 4, highlighted with an *asterisk* (*), was previously identified as a KLF2 site (46). *B,* KLF11 binds to site 3 of the *CXCR4* promoter *in vitro*. EMSA was performed using recombinant KLF11 protein (*lanes 1*, *3*, *5*, and *7*) or control GST protein (*lanes 2*, *4*, *6*, and *8*) with radiolabeled oligonucleotides for each of the four putative KLF binding sites, as indicated. Each probe was also loaded without protein (probe alone lanes, *a–d*). A KLF11/*CXCR4* oligo complex was significant with site 3 (*lane 5*), as indicated by the *arrow. C,* KLF11 binding to site 3 demonstrates binding specificity *in vitro*. EMSA was performed on WT *CXCR4* KLF site 3 (*WT site 3*; *lanes 1* and *3–8*) with GST protein (*lane 3*), recombinant KLF11 (KLF11; *lanes 2* and *4–8*), or probe alone (*lane 1*). Specific complexes between KLF11 and probe, as well as the free probe, are indicated by the *arrows* on the *left*. Although a GST antibody disrupted the KLF11/*CXCR4* WT site 3 complex (*lane 5*), the same amount of anti-mIgG did not (*lane 6*), indicating specificity. Excess unlabeled WT site 3 probe robustly competed for binding (×125 and 250; *lanes 7* and *8*, respectively), whereas excess mutant probe did not (×125; *lane d*). Radiolabeled mutant probe failed to bind recombinant KLF11 (*lane c*). Probe alone (*lane a*) and probe with GST protein (*lane b*) are shown as controls. *D,* functionality of the *CXCR4* KLF-HP1 site is provided by an intact site 3. Activity of the site 3 mutant *CXCR4* promoter was no longer derepressed by KLF11ΔHP1, indicating disruption of the KLF-HP1 site, whereas KLF11 WT showed negligible changes. Graphical depiction of the results is the mean ± S.E. from two independent experiments performed in triplicate.

Although previous studies have identified a KLF2 binding site in the *CXCR4* promoter (46), we analyzed the promoter for other potential KLF binding sites (Fig. 6*A*), and found 3 additional sites. To determine which site corresponds to the KLF11 binding site, we performed EMSA. Interestingly, KLF11 did not bind the KLF2 site (site 4; −173 to −178), suggesting preference for a different site on the *CXCR4* promoter. Specific binding of KLF11 was observed for site 3, corresponding to −119 through −129 of the *CXCR4* promoter (Fig. 6*B*). KLF11 binding to this sequence was found to be specific by complex disruption (Fig. 6*C*, *lanes 5* and *6*) and competition with cold WT but not mutant probe (Fig. 6*C*, *lanes 7*, *8*, and *d*). KLF11 did not bind to a radiolabeled probe mutated at site 3 (Fig. 6*C*, *lanes a-c*). We also performed site-directed mutagenesis on the *CXCR4* −300-bp reporter construct to change the corresponding site from CCGCCCCGCCCC to CTTTCCTTTCCC, which abolished derepression of the promoter by KLF11ΔHP1 (93.3% of control ± 19.1%, *p* > 0.05; Fig. 6*D*), consistent with a failure to recruit this KLF11 mutant. Collectively, these experiments identify the KLF site at −119 to −129 as the actual site that mediates HP1 recruitment to the *CXCR4* promoter in a sequence-specific manner. Due to the importance of HP1 recruitment in KLF11-mediated gene expression and the fact that KLF11 is a well established tumor suppressor, we subsequently investigated the significance of this mechanism for several cellular processes associated to this function.

##### HP1 Recruitment Is Required for KLF11-mediated Regulation of Cell Death, Proliferation, and Senescence

**FIGURE 7:**
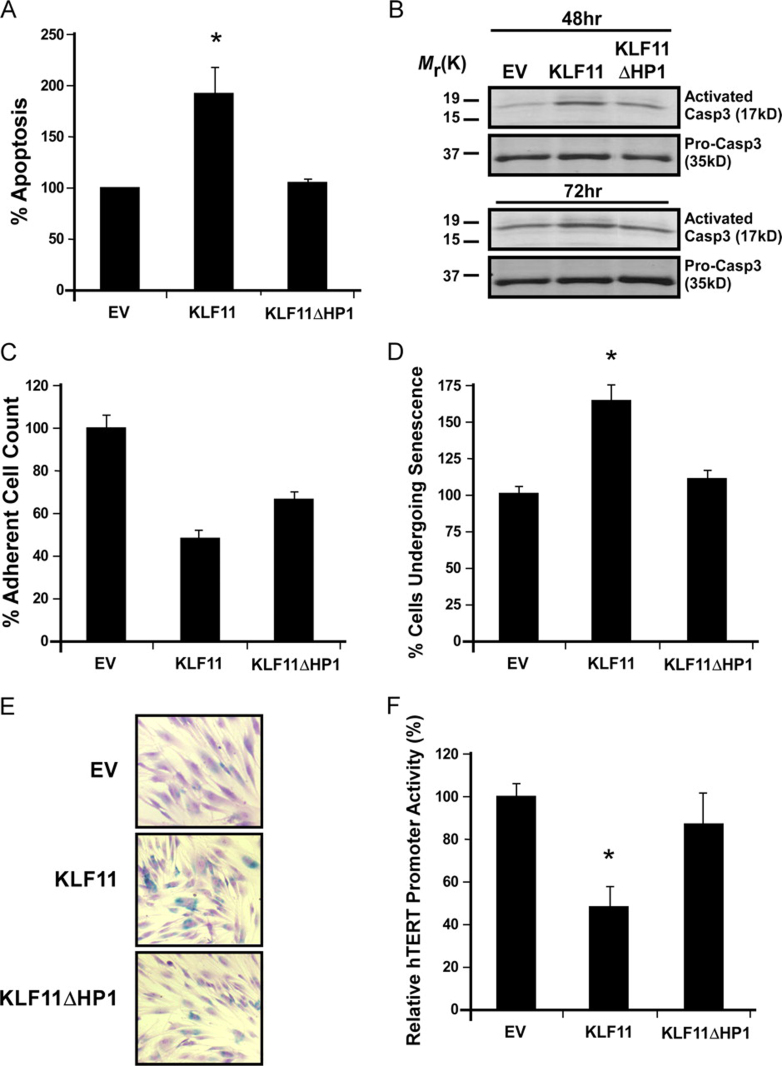
**HP1 recruitment is necessary for KLF11-mediated apoptosis and senescence.***A* and *B,* HP1 plays a role in the ability of KLF11 to induce apoptosis., Apoptosis was measured by both, nuclear morphology via Hoechst 33342 staining (*A*) and caspase 3 cleavage assay (*B*). As observed by Hoescht staining (*A*), KLF11 WT increases apoptosis. KLF11ΔHP1 is defective in inducing this effect, with an apoptotic index similar to EV. The depicted results are the mean ± S.E. from three independent experiments. In caspase 3 cleavage assays (*B*), KLF11 WT increased the amount of caspase 3 cleavage, as shown by Western blot (*upper*), whereas KLF11ΔHP1 had reduced levels similar to control. Pro-caspase 3 was used as a control (*lower*). The levels of caspase 3 cleavage are shown for both, 48- and 72-h post-serum starvation. *C*, KLF11ΔHP1 demonstrates an increase in adherent cell number compared with KLF WT. Assessment of adherent cell count is shown after normalization to EV. Although KLF11 WT results in a significant decrease in cell numbers, this effect is blunted with KLF11ΔHP1. The *graph* represents the mean ± S.E. of results from three independent experiments. *D* and *E,* loss of HP1 recruitment eliminates the ability of KLF11 to increase senescence. To measure cells undergoing senescence, senescence-associated β-galactosidase staining was performed in primary fibroblasts. KLF11 increased the percentage of cells undergoing senescence. However, KLF11ΔHP1 lost this ability, behaving similar to control. The *graph* (*D*) depicts the mean ± S.E. from three independent experiments. A representative picture of senescence-associated β-galactosidase staining for each experimental group is shown (*E*). *F,* KLF11ΔHP1 is no longer able to repress the telomerase promoter. Activity of the hTERT promoter, as measured by the luciferase reporter assay, was significantly repressed by KLF11 WT, however, KLF11ΔHP1 loses this repression. Graphical depiction of the results is the mean ± S.E. from at least three independent experiments performed in triplicate. * denotes *p* < 0.05.

Subsequently, we initiated investigations into whether the interaction between HP1 and KLF11 was necessary for KLF11-mediated tumor suppression, by measuring the rate of apoptosis and cell proliferation, two well known cellular mechanisms underlying this phenomenon (27). KLF11 WT increased apoptosis as demonstrated by nuclear morphology via Hoechst 33342 staining (Fig. 7*A*; 193.38 ± 25.55% normalized to EV, *p* < 0.05). In contrast, KLF11ΔHP1 disrupted the ability of this protein to induce apoptosis (106.19 ± 3.07% normalized to EV), displaying an apoptotic index similar to EV control. Caspase 3 cleavage assays, a marker of apoptotic pathway activation, demonstrated at both 48 and 72 h postserum starvation that KLF11 WT increased the amount of caspase 3 cleavage, whereas KLF11ΔHP1 had reduced levels similar to control (Fig. 7*B*). Additionally, the effect of KLF11-HP1α disruption on cellular proliferation was assessed by adherent cell count. KLF11ΔHP1 slightly increased cell proliferation as compared with KLF WT (Fig. 7*C,* 49 % ± 2.7% normalized to EV for KLF11 WT *versus* 66 ± 2.3% normalized to EV for KLF11ΔHP1, *p* < 0.05). Thus, the function of KLF11-mediated recruitment of HP1 is necessary to suppress cell growth via a significant effect on apoptosis and, to a lesser extent, proliferation.

We next evaluated the role of the KLF11-HP1 interaction in senescence. Senescence has been implicated *in vivo* as a tumor-suppression mechanism (47), and HP1 itself has been shown to participate in this phenomenon (48, 49). Furthermore, loss of HP1α has been associated to tumor progression (50). Notably, KLF11 increased the percentage of cells undergoing senescence by 62.5 ± 8.75% (Fig. 7, *D* and *E*), as shown via senescence-associated β-galactosidase staining in primary fibroblasts, normalized to control (*p* < 0.05). On the other hand, KLF11ΔHP1 impairs this ability, similar to the control (9.4 ± 5.3% above EV, Fig. 7, *D* and *E*). Congruently, luciferase reporter assays also show that whereas KLF11 WT repressed the telomerase (hTERT) promoter, a correlative surrogate for senescence (48.45 ± 7.87% of control; *p* < 0.05), KLF11ΔHP1 lost this activity (87.54 ± 13.48% of control) (Fig. 7*F*). Although, as mentioned above, this is not the first report of HP1 being involved in senescence, nevertheless, this is the first evidence that HP1 can also mediate this function in a sequence-specific manner, further supporting the function of this interaction in important cell biological functions associated to tumor suppression.

##### A Functional KLF11-HP1 Interaction Is Necessary for Suppression of Neoplastic Transformation and Tumorigenesis in Vivo

**FIGURE 8:**
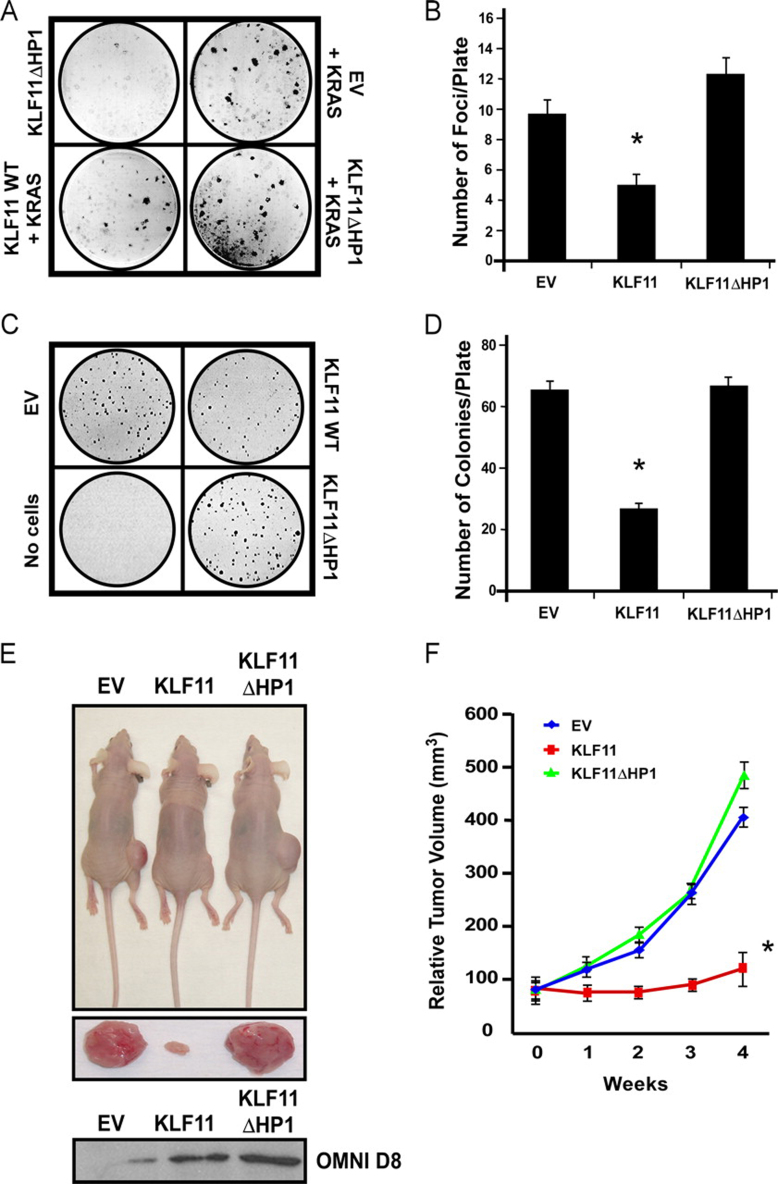
**HP1 recruitment is required for KLF11-mediated suppression of neoplastic cell growth.***A* and *B,* KLF11ΔHP1 is no longer able to suppress KRAS-mediated foci formation in NIH/3T3 cells. A representative photo (*A*) is shown for each: KLF11ΔHP1 alone (no KRAS), EV control + KRAS, KLF11 WT + KRAS, and KLF11ΔHP1 + KRAS. Note that KLF11ΔHP1 does not form foci alone in the absence of KRAS. Graphical depiction of the results (*B*) as the mean ± S.E. from three independent experiments performed at least in triplicate demonstrates that KLF11 WT significantly suppressed KRAS-mediated foci formation by 48%, whereas KLF11ΔHP1 failed to suppress KRAS-mediated foci formation. *C* and *D*, recruitment of HP1 is necessary for KLF11-mediated suppression of colony growth in soft agar. Panc1 cells were infected with adenovirus containing EV control, KLF11 WT, or KLF11ΔHP1 and monitored for visible colonies in soft agar. A representative photo is shown for each condition, including agar plated with no cells (*C*). KLF11 WT suppressed soft agar colony formation by ∼60%; however, KLF11ΔHP1 was unable to suppress colony formation. The graph (*D*) represents the mean ± S.E. from three independent experiments performed in triplicate. *E* and *F,* HP1 is necessary for KLF11-mediated tumor suppression *in vivo*. Flanks of athymic nude mice were injected with L3.6 pancreatic cancer cells transfected with control (*EV*), KLF11 WT, or KLF11ΔHP1. *E*, representative images of flank tumor burden (*upper*) and excised tumors at time of sacrifice (*middle*) are shown. Western blot with the OMNI D8 antibody confirms ectopic expression of His-tagged KLF11 WT and mutant proteins in injected cells (*lower*). *F,* graph depicts the mean tumor volume and S.D. at each week post-injection until sacrifice. KLF11 WT significantly decreases tumor size compared with control. However, KLF11ΔHP1 significantly impairs KLF11 tumor suppression, as seen in tumor sizes comparable with controls rather than KLF11 WT. * denotes *p* < 0.05.

Using foci formation assays, we evaluated the effect of the KLF11-HP1 interaction on anchorage-dependent cell growth of KRAS-transformed foci. KLF11 WT significantly suppressed KRAS-mediated foci formation by 48% (Fig. 8, *A* and *B*; mean 9.6 ± 0.97 colonies for EV *versus* 5.0 ± 0.73 for KLF11 WT, *p* < 0.05). However, disruption of HP1 recruitment no longer suppressed neoplastic transformation (127% normalized to EV; mean 12.3 ± 1.1 colonies). KLF11ΔHP1 reversed KLF11 suppressive activity but did not significantly enhance KRAS-mediated transformation nor was oncogenic alone in this assay. We next tested impact of the KLF11-HP1 interaction on KLF11-mediated suppression of anchorage-independent cell growth in soft agar assays. Consistent with foci formation, KLF11 WT suppressed agar colony formation by ∼60% (mean 65.8 ± 2.3 colonies for EV *versus* 26.8 ± 1.6 for KLF11 WT, *p* < 0.05), whereas KLF11ΔHP1 failed to suppress agar colony formation (Fig. 8, *C* and *D*; mean 67.3 ± 3.1 colonies). Collectively, these results demonstrate that HP1 is a necessary cofactor for the suppression of both, anchorage-dependent and -independent cell growth when being recruited in a sequence-specific manner.

We also performed tumorigenesis assays *in vivo*, injecting flanks of athymic nude mice with L3.6 pancreatic cancer cells transfected with EV, KLF11 WT, or KLF11ΔHP1 (Fig. 8*E*). Notably, whereas KLF11 WT decreased tumor size compared with control (133.5 mm^3^ ± 25.9 mean tumor volume after 4 weeks for KLF11 WT *versus* 405.3 mm^3^ ± 16.6 for EV, *p* < 0.05), failure of HP1 recruitment (KLF11ΔHP1) impaired tumor suppressive activity (Fig. 8, *E* and *F*; 478.2 mm^3^ ± 24.1 mean tumor volume after 4 weeks). Similar results were obtained using Panc1 cells (data not shown). Combined, these comprehensive data reveal a novel role for HP1 as a cofactor of KLF11 in tumor suppression, define distinct cellular mechanisms underlying these effects, and illustrate that this KLF11-mediated mechanism of sequence-specific HP1 recruitment has significant biological and pathobiological consequences.

## DISCUSSION

Here, we report several novel findings that advance our understanding on how chromatin functions to regulate gene expression, including the first description of direct sequence-specific recruitment of HP1 rather than its binding to H3K9me. Our work defined interaction between HP1 and the tumor suppressor protein and sequence-specific transcription factor, KLF11. Mapping of this HP1 binding site to the KLF11 C-terminal domain generated a specific deletion mutant, KLF11ΔHP1. Importantly, this mutant was able to still bind KLF sites within promoters and complex with other proteins that are known to act as cofactors for KLF11 transcriptional activity, but failed to recruit HP1, thereby furthering our mechanistic experiments. Biochemically, we show that KLF11 regulates the expression of *CXCR4* at a specific KLF11 binding site (−119 to −129), which functions to recruit HP1 as well as its associated HMT, SUV39H1, confirming that this KLF site ultimately works to recruit HP1-HMT complexes to the promoter. Functional KLF11-HP1-SUV39H1 recruitment is associated with an increase of H3K9me3 marks, which did not occur in the absence of HP1 recruitment by KLF11ΔHP1, indicating that the H3K9me3 mark does not precede HP1 recruitment in this context. Thus, the function of this mechanism is to direct recruitment of HP1-HMT complexes in a manner that is similar to the polycomb PRE, demonstrating some mechanistic conservation between these related HMT systems. HP1 triggers this function by being recruited to promoters in a sequence-specific manner rather than its well characterized binding to methylated chromatin, indicative of how this system can function in the regulation of gene expression with a higher degree of specificity.

**FIGURE 9:**
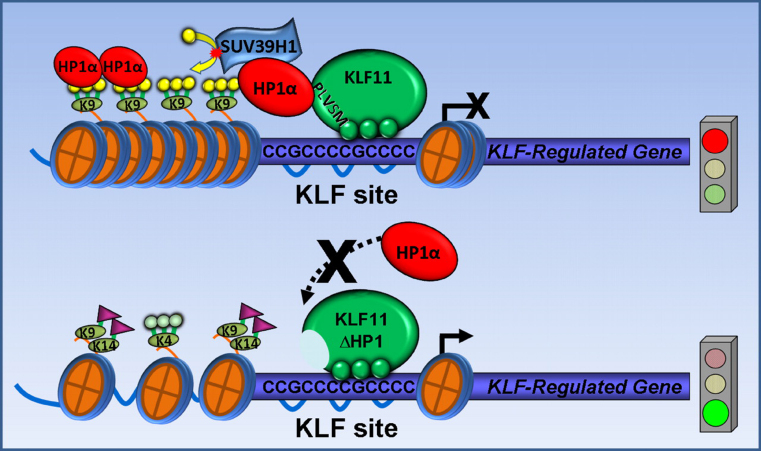
**Model of HP1 recruitment in KLF11-mediated gene regulation.** An extended KLF11 binding site mediates the sequence-specific recruitment of HP1-SUV39H1 to regulate gene promoters (*upper*). Our experimental data demonstrate that engagement of HP1-SUV39H1 recruitment by KLF11 binding leads to the deposition and/or removal of key histone marks that change the state of chromatin and impact on transcription. Disruption of this recruitment mechanism, as achieved by KLF11ΔHP1, leads to derepression of gene promoters (*lower*).

Notably, elegant studies have previously demonstrated KRAB (Krüppel-associated box)-containing zinc finger transcription factors recruit KAP1 (KRAB-ZFP associated protein 1), which binds to HP1 (51, 52). Krüppel-associated box zinc finger proteins, however, do not bind directly to HP1, as shown here for KLF11, but rather recruit this protein via a transcriptional intermediary, KAP1. Interestingly, disruption of the P*X*VXL-mediated interaction of HP1 with KAP1 triggers the dissociation of this transcriptional intermediary protein from target promoters (53, 54). On the other hand, KLF11 binds to its target DNA sequence regardless of whether it is bound or not to HP1. The two obvious differences indicate that the biophysical and biochemical properties that regulate these two mechanisms for recruiting HP1 to promoters are readily distinct. Due to its mechanistic importance, we have integrated the results of this study into a model (Fig. 9). An extended KLF11 binding site (CCGCCCCGCCCC) mediates the sequence-specific recruitment of HP1-SUV39H1 to promoters. The adjacency of nucleosome recognition sequences suggests that this recruitment would in turn modify histones. Indeed, our experimental data demonstrate that engagement of this site by KLF binding leads to the deposition and/or removal of key histone marks that change the state of chromatin and impact on transcription. In our gene model (*CXCR4*), the KLF11-HP1 interaction appears to limit the activation achieved by histone acetyltransferases, as well as the deposition of activation histone marks. Disruption of this mechanism, as achieved by KLF11ΔHP1, leads to deregulation of this gene (derepression). This event is accompanied by the reversal of H3K9me3 to acetylation of this residue and H3K14, along with the maintenance of the activating H3K4me3 mark, congruent with a derepressed promoter state. Together, this information supports the idea that an important function of the KLF11-HP1 interaction is to maintain gene expression within physiological levels by offering a default “brake” limiting the histone acetyltransferase-mediated activation of promoters.

Interestingly, Sp/KLF sites have been previously described as part of the sequence-specific recruitment of the polycomb-HMT system, with these sites being present in almost all known *Drosophila melanogaster* PREs and a specific Sp/KLF binding site required for activity of the 181-bp *engrailed* PRE (22). A recent study describes that at least one member of the KLF family, Spps, is critical to the regulation of this KLF-PRE (23). Similar to the KLF-HP1 recruitment site described here, the *Drosophila* KLF-PRE has binding sites for other transcription factors, indicating that additional combinatorial effects, besides those critically mediated by KLF proteins, may modulate the recruitment of both HMT systems to promoters. Interestingly, as described for the KLF-PRE mutation of the KLF site in the *Drosophila engrailed* gene (22), the KLF site described here is critical to the transcriptional activity and chromatin remodeling events of the *CXCR4* promoter, as well as cell functions associated with KLF11-HP1 interaction. Overall, the discovery of this KLF11-mediated HP1 recruitment, together with the knowledge of *Drosophila* KLF-PREs, suggest that KLF sites have undergone evolutionary pressure to maintain their ability to recruit potent HMT-based silencing complexes, likely changing their selectivity for each complex during this process.

HP1 has been implicated in cancer (55, 56, 57), although remains poorly understood at the mechanistic level. Disrupting KLF11 recruitment of HP1 impairs its ability to inhibit anchorage-dependent and -independent cell growth and *in vivo* tumorigenesis, as well as its ability to increase apoptosis and senescence. Collectively, these results demonstrate that HP1 binds to a sequence-specific tumor suppressor protein and provides the best characterized evidence for participation of HP1 in binding and executing the function of a tumor suppressor and sequence-specific transcription factor to antagonize neoplastic transformation.

The current study also extends our understanding of novel molecular mechanisms mediating the function of KLF proteins. The observation that KLF11 can work via histone acetylation (p300), deacetylation (Sin3a), and histone methylation (HP1), as described here, indicates that this sequence-specific transcription factor and tumor suppressor has many functions that occur via different pathways or alternatively, that several pathways are required for the same function. In addition, it is interesting to consider the convergence of short-term (Sin3a) and long-term (HP1) repression mechanisms on the same molecule (KLF11). Thus, studies that identify novel cofactors for KLF proteins, as described here for HP1-KLF11, will continue to extend our understanding of the molecular mechanisms underlying the function of this important family of proteins, which regulate a myriad of functions in most tissues and organisms.

In conclusion, the current study identifies and characterizes a P*X*V*X*(L/M) domain within KLF11, which functions to directly recruit HP1-HMT complexes to promoters. Biologically, this HP1-HMT recruitment is required for KLF11-mediated gene regulation and tumor suppression. Thus, this data significantly advances our understanding of how chromatin-mediated mechanisms achieve these functions with increased specificity for target genes.

## Footnotes

3 The abbreviations used are:

HP1: heterochromatin protein 1
KLF: Krüppel-like factor
PRE: polycomb response element
BiFC: bimolecular fluorescence complementation
EV: empty vector
HMT: histone methyltransferase.
