# Supplementary material for: Sequence-specific Recruitment of Heterochromatin Protein 1 via Interaction with Krüppel-like Factor 11, a Human Transcription Factor Involved in Tumor Suppression and Metabolic Diseases
Source: J Biol Chem. 2020 Dec 18;287(16):13026–39. doi: 10.1074/jbc.M112.342634 (PMC3339955; doi:10.1074/jbc.M112.342634)
Supplement: Supplementary file 1 [file mmc1.zip › jbc.M112.342634-2.pdf]

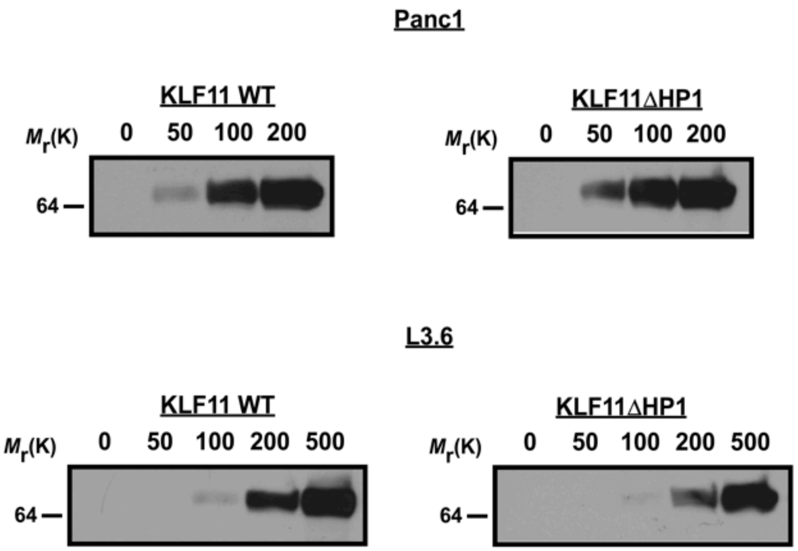

Supplementary Figure: Ectopic expression of His-tagged KLF11 WT and KLF11ΔHP1 proteins. Multiplicity of infection (MOI) curve is shown for Panc1 and L3.6 cells to confirm expression of KLF11 WT and KLF11ΔHP1 mutant proteins.
